# Self-care behavior: a new insight of the role of impulsivity into decision making process in persons with heart failure

**DOI:** 10.1186/s12872-020-01617-8

**Published:** 2020-07-27

**Authors:** Mohammed Munther Al-Hammouri, Jehad A. Rababah, Lynne A. Hall, Debra K. Moser, Zainab Dawood, Wa’ed Jawhar, Ayat Alawawdeh

**Affiliations:** 1 Department of Community and Mental Health Nursing, Faculty of Nursing, Jordan University of Science and Technology, P.O.Box 3030, Irbid, 22110 Jordan; 2 Adult Health Nursing Department, Jordan University of Science and Technology, Irbid, Jordan; 3 School of Nursing, University of Louisville, Louisville, USA; 4 School of Nursing, University of Kentucky, Lexington, USA; 5 Jordan University of Science and Technology, Irbid, Jordan; 6 King Hussien Cancer Center, Amman, Jordan

**Keywords:** Heart failure, Self-care behavior, Impulsivity, Decision making, Nursing

## Abstract

**Background:**

Self-care behavior has been reported to be below optimum in persons with heart failure, while the underlying decision making is not well understood. The Hot/Cool System model is a psychological model that may have potential applications in decision making process in persons with heart failure. The aim of this study was to examine the decision making process in self-care behavior in persons with heart failure in the light of the Hot/Cool System model.

**Methods:**

We used the Hoot/Cool System Model to guide this study. Participants with heart failure from in-patients setting (*N* = 107) were recruited. Data were collected using self-report questionnaires. Moderated mediation analysis was used to study complex relationships among study variables.

**Results:**

The current study showed that impulsivity and perceived stress were negatively associated with self-care behavior. The results also showed that self-care confidence and impulsivity significantly predict self-care maintenance. The moderated mediation analysis revealed that self-care confidence mediated the relationship between impulsivity and self-care maintenance at lower levels of perceived stress, but not at higher levels of perceived stress.

**Conclusion:**

Our findings revealed that persons with heart failure tend to make impulsive choices that may negatively affect disease progression under higher levels of perceived stress. This study provides foundational knowledge regarding the decision making process in persons with heart failure.

## Background

Heart failure (HF) is one of the most debilitating conditions affecting individuals worldwide, associated with frequent hospitalization and high mortality and morbidity rates [1]. Lifelong behavioral changes are recommended to improve future outcomes, such as HF progression, admission and readmission, through optimal self-care behavior [2–4]. Many investigators have explored self-care behavior in persons with HF. Better self-care behavior has been associated with higher education levels, lower symptom severity, less depression, and greater self-care [5–7]. In these studies, the variance in self-care behavior was only partially explained [5, 8], and complex relationships among predictors have rarely been investigated [5–7]. In addition, the underlying decision-making process in a person with HF is still unclear [9].

To study the decision-making process in a person with HF, there is a need to examine new self-care behavior in persons with HF. That need has emerged for two reasons. First, we need to find new potential predictors that may more fully explain self-care behavior among persons with HF. Second, we need to study the complex interrelationship among predictors of self-care behavior as a decision-making process. In this study, we investigated the interrelationships among three factors that predicted decision-making in prior research, although not in HF: impulsivity, self-care confidence, and perceived stress.

**Fig. 1:**
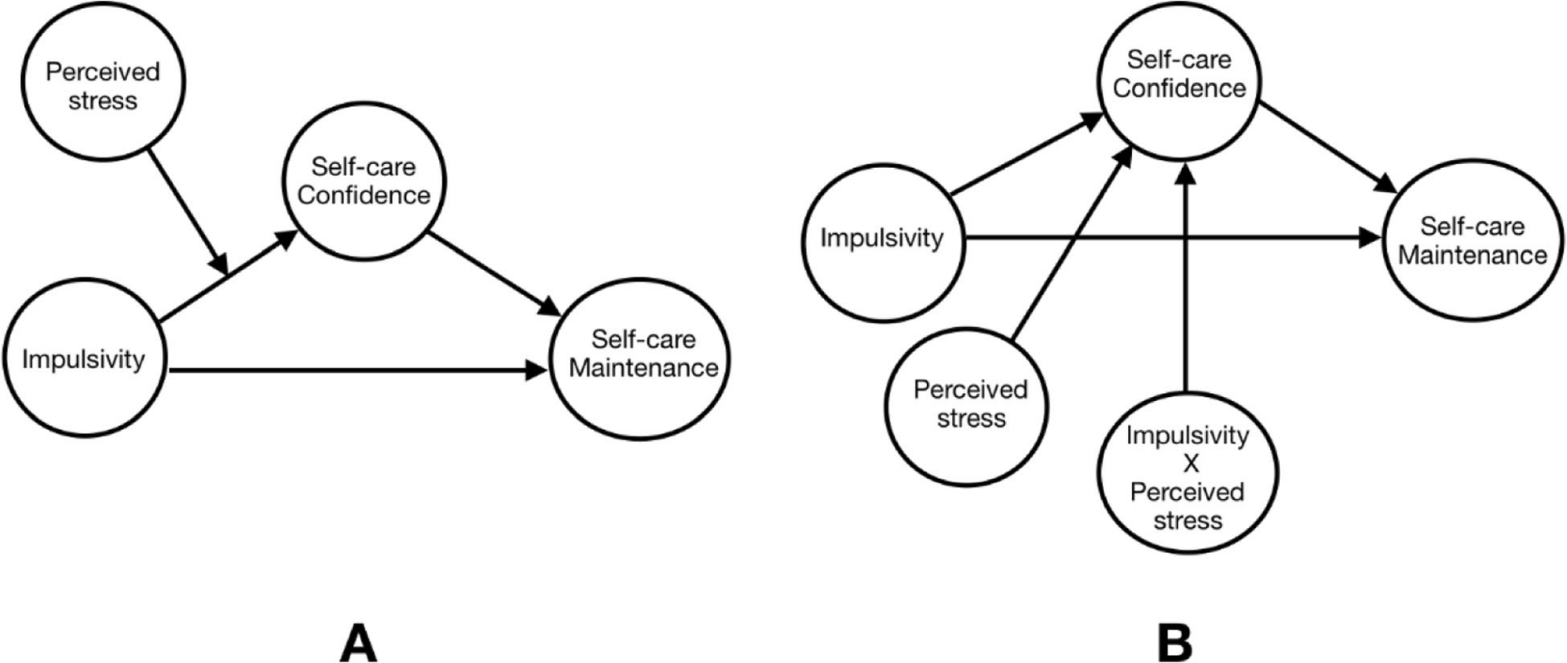
**a** Theoretical model adopted from Metcalfe and Mischel (1999) and Mischel (2014) [10, 11]. **b** Statistical model adopted from model 2 Hayes (2013) [12]

The Hot/Cool System Model, a model developed in psychology, guided the current study. The model proposes a process to explain and predict how internal and external factors interact to determine the course of action a person may take when the person is given a choice to act in many different ways. According to the model, presenting a person with a choice-making situation will initially trigger the hot system [10, 11], which can be considered as the emotional component in the model. The activated hot system tends to make the individual more reflexive, rapid, and emotional in making choices. The hot system in the current study is represented by impulsivity (see Fig. 1). As we will discuss later, impulsivity represents the part of our behvaior that is reflexive and emotional. The activated hot system, in turn, activates corresponding areas in the cool system [10, 11]. This part of the system is the cognitive part of the system, which helps the person to be more reflective, self-controlled, and take a more responsible course of action. In the current study, self-care confidence represented the cool system. Self-care confidence reflects person’s ability to detect and interpret the meaning of HF symptom that involve cognitive processing. The outcome behavior is determined by whichever of the two systems is dominant at the time of making a choice. However, the dominance of one system over the other (Hot system versus cool system) can be affected by contextual variables [10, 11].

One proposed contextual variable discussed by Metcalfe and Mischel is stress (Fig. 1) [ 10]. According to the Hot/Cool System, the hot system tends to be the dominant system in the case of high stress, as stress empowers the effect of the hot system and attenuates the effect of the cool system. In the case of low stress, the cool system is empowered, and the hot system is attenuated, which makes the cool system more powerful than the hot system. To the best of our knowledge, this model has not been used to investigate health-related behaviors, such as self-care behavior in chronic illnesses in general and HF in specific. In this study, perceived stress was used to measure stress in persons with HF (Fig. 1). The Hot /Cool System Model was proposed to explain the findings of previous research on delayed gratification [10]. Research on delayed gratification studies the effect of the temporal delay of the consequences of our behaviors on controlling future reoccurrence of those behaviors.

In the current study, we used this model to explore variations in self-care behavior among persons with HF (Fig. 1). The model may help us determine why persons with HF often make self-care decisions that seem counter to their best interests. When depicting our study variables on the adopted model, impulsivity was used to assess the relative strength of the emotional component of the model (i.e., the hot system), and self-care confidence was used to assess the relative strength of the cognitive component of the model (i.e., the cool system). As proposed by the model, perceived stress is a third variable that can shift the dominancy from one system to another. The goal of this study was to examine the decision-making process in self-care behavior in persons with HF in light of the Hot/Cool System model. Specifically, the current study was conducted to evaluate whether self-care confidence would differentially mediate the relationship between impulsivity and self-care behavior at different levels of perceived stress in persons with HF, as proposed by the Hot/Cool System Model.

## Methods

### Design and setting

This study was a cross-sectional exploratory study. Data were collected from eligible participants using self-report questionnaires during their hospital stay at University hospital, which is considered the referral hospital in northern Jordan. Institutional Review Board approval was obtained from the host university, and written informed consent was obtained from each participant prior to the data collection. The data were collected by research assistants who were trained in recruiting and interviewing participants in the current study. The research assistants were nurses working in the hospital wards, where persons with HF were admitted.

### Sample

A purposive sample of hospitalized persons with HF was recruited. The inclusion criteria were a diagnosis of HF for 6 months, at least 18 years of age, and able to read and write to fill out self-report questionnaires. Exclusion criteria were a diagnosis of dementia and co-existing terminal illnesses. Those with terminal illnesses usually suffer serious health issues, and asking them to participate in the current study was not appropriate. This decision was based on the research assistants’ anecdotes after starting the data collection. The diagnosis of HF was determined by checking the person’s diagnosis, as indicated by the electronic record. In addition, the reason for admission was specified to be related to the exacerbation of HF symptoms. One hundred and seven participants completed and returned the study questionnaires. The sample size was determined based on a conservative, small-medium expected effect size using multiple linear regression to test the mediational relationship. Impulsivity and its relationship to self-care behavior have not been studied in prior research. Thus, there were no available prior reports to determine the expected effect size. Cohen reported that there are three main levels of effect sizes: small (0.02), medium (0.15), and large (0.35) [13]. Thus, a sample of 104 persons with HF based on a small to medium effect size (0.11) and power of 0.80 was deemed sufficient for evaluating the proposed relationships.

### Measures

The level of self-care maintenance and self-care confidence was determined using self-care maintenance and self-care confidence subscales, respectively, from the Self-Care of Heart Failure Index, Version 6.2 (SCHFI-V6) [14]. The self-care maintenance subscale in the SCHFI-V6 consists of 10 items with a 4-point Likert-like response scale [14]. The self-care confidence subscale in the SCHFI-V6 consists of six items with a 4-point Likert-like response scale [14]. Individual subscales scores were used in the analysis as recommended by Riegel and colleagues [14]. Scores were standardized by converting each subscale score to a 100-point scale for ease of comparisons among different subscales, different studies, and different versions of self-care measures. Higher scores reflect better levels of self-care behavior. A cutoff score of 70 out of 100 defines adequate self-care behavior. The reliability and validity of the SCHFI-V6 have been examined and found adequate [14]. Cronbach’s α in the current study were .61 and .72 for the self-care maintenance and self-care confidence scales, respectively.

The level of impulsivity of persons with HF was assessed using the Barrett Impulsiveness Scale (BIS-11). The BIS-11 is the most widely used measure of impulsivity [15]. It consists of 30 items with a 4-point Likert-like scale ranging from 1 (Rarely/Never) to 4 (Almost Always) and is divided into three subscales: non-planning impulsivity (11 items); motor impulsivity (11 items); and attention impulsivity (8 items). The total BIS-11 score was used as an indicator of the level of impulsivity in the current study. Stanford and colleagues suggested the following categorization of total scores: 72 or above as high impulsivity, 52–71 as normal impulsivity, and 30–51 as over-control [15]. The BIS-11 is available in 11 languages. According to Stanford and colleagues, all translated versions have acceptable internal consistency: Cronbach’s alphas ranged from .71 to .83 [15]. Stanford et al. reported internal consistency of .83 and Spearman’s Rho for one-month test-retest reliability of .83 in a sample of adults [15]. Cronbach’s α of the BIS-11 was .66 in the current study.

Perceived stress was assessed using the Perceived Stress Scale-10 (PSS) [16]. The PSS-10 is composed of 10 items rated on a 5-point Likert-like response scale from 0 (never) to 4 (very often). The total score ranges from 0 to 40. The higher the score, the higher the level of perceived stress. Lee (2012) reviewed the psychometrics of the three versions of the PSS (PSS-4, PSS-10, and PSS-14) and showed that PSS-10 has the best psychometrics among the three versions, while the PSS-4 has the worst [17]. Thus, the PSS-10 was used in this study. Cronbach’s alpha for the PSS-10 was above .70. Lee found that test-retest reliability for PSS-10 was assessed in four studies and was acceptable (above .70) [17]. The results of this study showed that the Cronbach’s α was .75 for the PSS-10.

### Covariates

Based on the revision of the related literature, three covariates were added. These are depression, functional status, and HF knowledge, and the literature shows that they all play a key role in determining the self-care behavior of persons with HF. Depression is a major determinant of health-related quality of life in persons with HF [18]. Depression was evaluated using the Patient Health Questionnaire–9 (PHQ-9) [19], which is composed of nine items that ask about the frequency of problems patients suffered in the last 2 weeks. The response options for those questions range from 0 “not at all” to 3 “nearly every day.” The total score ranges between 0 and 27; the higher the score, the more severe the level of depression. According to Kroenke et al., scores of 5, 10, 15, and 20 on the PHQ-9 represent mild, moderate, moderate to severe, and severe levels of depression, respectively [19]. The psychometric properties of PHQ-9 were examined with a sample of 322 persons with HF [20]. The PHQ-9 had good internal consistency with Cronbach’s alpha of .83. Inter-item correlations ranged from .22–.66 [20].

Functional status was assessed using the New York Heart Association (NYHA) functional classification [21]. The NYHA class is determined by the occurrence of fatigue, dyspnea, angina, or palpitations with different levels of physical activity. The NYHA class ranges from I (no symptoms with ordinary physical activity) to IV (symptoms occur at rest). The construct validity of the NYHA was supported in different ways. For example, the agreement between the NYHA and Four Weber classifications of the exercise test was 41.7% (*p =* .005) [22]. In addition, the NYHA class was concordant with the 6-min walk test in 42% of patients (*p* = .001) [22]. Goldman, Hashimoto, Cook, and Loscalzo (1981) assessed the inter-observer reliability of the NYHA where the agreement was 56% between cardiologists and patients’ physicians [22].

HF knowledge was assessed using the Atlanta Heart Failure Knowledge Test Version 3 (AHFKT). The AHFKT consists of 30 questions about several domains in HF knowledge: pathophysiology, nutrition, medications, behavior, and HF symptoms [22]. The total score can be obtained by counting the number of correct answers. The score ranges between 0 and 30. The higher the score, the better the knowledge about HF. Cronbach alpha values were .84 for persons with HF, and .75 for family members of persons with HF [23]. Construct validity indicates that higher HF knowledge is associated with better self-care behavior in persons with HF [23]. Simple Demographic data were collected using a questionnaire developed by the authors of this study (see Supplementary File 1).

### Data analysis

The data were analyzed using SPSS® version 25 (IBM, Armonk, NY). Mean, median, range, and standard deviations of the continuous variables and frequencies for categorical variables were used to address sample characteristics and look for any potential problems with the data. Mean imputation was used to replace missing data because the rates of missing data for all of them were less than 2% for all variables. The assumption was tested before carrying out the analysis.

Initially, bivariate relationships among study variables were tested based on Baron and Kenny’s guidelines for testing moderation and mediation relationships [24]. We tested the proposed moderated mediation model using regression-based SPSS macros developed by Hayes [12]. Based on that model, we tested two levels of relationships. At the first level, the mediation relationship was tested to determine if entering self-care confidence as a mediator would affect the direct relationship between impulsivity and self-care maintenance.

At the second level, we tested the moderation effect of perceived stress on the previously mentioned mediational relationship. We tested the moderation to check if self-care confidence would differentially mediate the effect of impulsivity on self-care behaviors at different levels of perceived stress based on the Hot/Cool System Model. Finally, we used bootstrapping to test if that effect was significant.

## Results

### Sample characteristics

The mean age of the sample was 58.5 years (*SD* = 11.7). The participants ranged in age from 31 to 87 years. The demographic and clinical characteristics of the sample are displayed in Table 1. According to Stanford and colleagues’ categorization [15], the largest category for impulsivity in the current sample was the normal impulsivity group (*n* = 74), followed by the high impulsivity group (*n* = 27), and finally by the over-controlled group (*n* = 6). According to Kroenke and colleagues’ categorization for the PHQ-9 [20], the majority of the sample (*n* = 94) had scores of 5 and above, indicating mild to severe depression. The results showed that 103 participants out of the 107 participants had below optimal self-care maintenance.

**Table 1:** Demographic and Clinical Characteristics of the Study Sample (*N* = 107)

Variable		*n*	~%
Sex	Female	23	21.5
	Male	84	78.5
Smoking	Smoker	42	39.0
	Non-smoker	65	61.0
Education	Did not complete high school	48	45.0
	High school diploma	30	28.0
	Vocational or some college	13	12.0
	College	16	15.0
Functional status	Class I	8	7.5
	Class II	44	41.0
	Class III	39	36.5
	Class IV	16	15.0

Our dependent variable, self-care maintenance, was positively correlated with self-care confidence (*r* =. 0.40, *p* < .001) and HF knowledge (*r* = .29, *p* < .01). Self-care maintenance was negatively correlated with impulsivity (*r* = −.23, *p* < .05) and perceived stress (*r* = −.26, *p* < .001) but not with depression and HF knowledge. One-way ANOVA showed that mean self-care maintenance did not differ by functional status class.

In the first step of testing the proposed model, self-care confidence was regressed onto impulsivity, perceived stress, HF knowledge, functional status, depression, and the interaction between impulsivity and perceived stress. The significant variables in this model were perceived stress, impulsivity, and the interaction term between impulsivity and perceived stress (Table 2). This model was not significant (*F* (6, 100) = 1.98, *p* = .07). In the next step, we regressed self-care maintenance onto impulsivity, self-care confidence, perceived stress, HF knowledge, functional status, and depression (Table 2). The model explained about 28% of the variance in self-care maintenance (*F* (5, 101) = 7.73, *p* < .001). In this model, the significant variables were impulsivity, self-care confidence, and HF knowledge.

**Table 2:** Regression Results for self-care confidence and Self-care Maintenance Models (*N* = 107)

Dependent variable	Predictor	*B*	SE	*t*	*p*
Self-care Confidence					
Constant		165.78	44.63	3.71	.00
Impulsivity		−1.62	.68	−2.37	.01
Perceived stress		−4.99	2.16	−2.30	.02
Impulsivity X Self-care confidence		.06	.03	2.07	.04
Heart failure knowledge		.29	.43	.66	.50
Functional status		−2.13	2.44	−87	.38
Depression		.53	.36	1.48	.14
Self-Care Maintenance					
Constant		46.49	14.43	3.22	.00
Self-care confidence		.27	.07	3.88	.00
Impulsivity		−.37	.16	−2.27	.02
Heart failure knowledge		−.73	.31	2.31	.02
Functional status		−3.00	1.74	−1.72	.08
Depression		.08	.23	.34	.73

The Hayes process was used to test the moderation effect of perceived stress on the indirect relationships between impulsivity and self-care maintenance. This process tests the indirect relationships at three different levels of perceived stress. These levels are + 1 SD, mean, − 1 SD for high, moderate, and low levels of perceived stress, respectively (Table 3). These categories represented low (− 1 SD), moderate (the mean), and high (+ 1 SD) perceived stress levels. The direct relationship between impulsivity and self-care maintenance was significant (Table 3). The indirect relationship between impulsivity and self-care maintenance through self-care confidence was only significant at the low level of perceived stress (Table 3). The index of moderated mediation shows that the moderated mediation relationship proposed in our model is significant, which means that perceived confidence only mediated the effect of impulsivity on self-care maintenance at lower levels of perceived stress as proposed by the Hot/Cool System model.

**Table 3:** Regression Results for Conditional Direct and Indirect Effects of Impulsivity on Self-care Maintenance (*N* = 100)

Direct effect of impulsivity on self-care maintenance				
Effect	SE	*t*	*p*	95% CIs
-.37	.16	−2.27	.02	−.6945, −.0470
Conditional indirect effect of impulsivity on self-care maintenance at perceived stress score = +/−1 SD				
Perceived stress	Effect	Boot SE	Boot 95% CIs	
-1 SD	−.19	.09	−.4040, −.0297	
Mean	−.06	.06	−.2110, .0626	
+ 1 SD	.06	.09	−.1148, .2739	
Index of Moderated Mediation				
	Effect	Boot SE	Boot 95% CIs	
Perceived stress	.01	.01	.0012, .0395	

*CIs* confidence intervals

## Discussion

The aim of the current study was to explore self-care behavior as a decision- making process in light of the Hot/Cool System Model. The best way to achieve this aim was to study how complex relationships among interacting variables affect self-care behavior in persons with HF. In addition to guiding the analysis of the complex relationship among study variables, the Hot/Cool System Model introduced impulsivity as a potential predictor of self-care behaviors in persons with HF.

We demonstrated that 97% of our sample had sub-optimal self-care behavior. These results were consistent with previous literature [14]. Our sample had a high level of perceived stress, which is consistent with the literature when compared with the norm of the same age group [25]. We also found that 88% of our sample suffered from mild to severe depression that is consistent with the high prevalence of depression in persons with HF. Impulsivity and perceived stress were negatively associated with self-care behavior, which was consistent with what is proposed in the literature. We expected that depression would have a significant, negative association with self-care maintenance in the regression model, as proposed by the literature. However, when impulsivity was entered into the model (Table 2), depression was not associated with self-care maintenance. This could be due to the effect of impulsivity that masked the effect of depression when entered into the model. In other words, the role of impulsivity in determining self-care maintenance is more prominent than the role of depression.

We proposed that self-care confidence would differentially mediate the relationship between impulsivity and self-care maintenance as a measure of self-care behavior at different levels of stress. This study proposition was based on the Hot /Cool System Model [10]. Initially, self-care maintenance was significantly associated with self-care confidence, impulsivity, perceived stress, and HF knowledge, but not with depression. Thus, the moderated mediation model proposed was supported; self-care confidence moderated the effect on impulsivity on self-care maintenance at lower level of stress.

These results are significant in three main ways. First, the Hot/Cool system Model was helpful in predicting complex relationships among the current study variables. Despite its usefulness, we could not locate any study in the health literature that used this model to investigate self-care behavior in persons with HF. Second, the model helped in proposing a new predictor for the first time, i.e., impulsivity, to the self-care behavior in HF. We believe that introducing new variables to help to predict self-care behavior in persons with HF will help us understand the mechanism by which we may help those persons to control their own health. The same may apply to other chronic illnesses, such as diabetes mellitus, hypertension, and other cardiovascular diseases.

Finally, the current study opened the door for the use of behavioral interventions to promote self-care behavior in persons with HF. Impulsivity, according to the results reported here, is a possible target for in-depth investigation of the effectiveness of behavioral interventions that target impulsivity and its effect on the self-care behavior of persons with HF. A variety of interventions have been used to influence the level of impulsivity. Those interventions were used in other fields, such as psychology, for behavior modification purposes. Examples of those interventions are framing, priming, reward bundling, brain training, and contingency management that can be part of a behavior modification plan to promote self-care behavior in persons with HF [26, 27]. For example, brain training is an intervention that makes use of active participation in mental processes that counter the effect of impulsivity on an intended behavioral outcome [26]. This intervention is directed toward the use of variables associated with the cool system variables proposed by the Hot/Cool System Model. Contingency interventions, on the other hand, add artificial contingencies to the maladaptive behavioral choice to make it less tempting to the person to reduce the emotional reaction to the immediate consequences [27]. This intervention focuses on the emotional part of the Hot/Cool System Model (i.e., the Hot system). A different area to be targeted by behavioral intervention could be stress reduction techniques, which, according to the current study, would help to potentiate the cool system and help the person toward making self-controlled decisions and promoting self-care behavior. However, the clinical benefits of these interventions require further research to test their efficiency in promoting self-care behavior.

The study results are limited by the sampling approach used. Random sampling could improve the rigor of future investigation. The current sample was also limited to hospitalized persons with HF. The study was conducted at a single setting, as permission for data collection from other settings was not obtained prior to the beginning of the current study, due to administration issues related to those sites. The results of the current study have limited generalizability, as the relationships studied here, the model used in this study, and impulsivity as a predictor of self-care behavior in persons with HF was used for the first time in the self-care literature in chronic illnesses. Thus, systematic replication is warranted. In addition, the use of SCHFI posed a problem in scoring self-care management in persons with HF. With half of our sample missing the self-care management scores, we could not incorporate self-care management as a measure of self-care behavior in persons with HF. Thus, replicating the study with a larger sample size is warranted to probably overcome this issue. In addition, using sample with various racial and demographic characteristic is needed to support the results of current study.

## Conclusion

In summary, the results supported what was proposed by the Hot/Cool System Model. These results provided new insights about how impulsivity and self-care confidence may interact to affect self-care maintenance in persons with HF. However, such interactions are affected by perceived stress that changes the level of self-care maintenance in persons with HF. Such interaction between the studied variables can be applied to persons with HF to improve self-care behavior. Thus, healthcare providers are encouraged to address such complex interaction in determining the self-care behavior of persons with HF. The current research can be considered a step toward facilitating the adoption of behavioral research finding in practice in compliance.

## Supplementary information

**Additional file 1.** Simple demographic questionnaire was developed by the authors of this study to collect basic demographic data of the participants.

## Data Availability

The datasets used and/or analyzed during the current study are available from the corresponding author on reasonable request.

## Abbreviations

ACT: Acceptance and commitment therapy
AHFKT: The Atlanta heart failure knowledge test
BIS-11: Barret impulsiveness scale
CAS-R: The control attitudes scale-revised
CI: Confidence interval
F: F-test
HF: Heart failure
NYHA: New York heart association classification
P: *P* value
PHQ-9: The patient health questionnaire–9
PSS: The perceived stress Scale-10
r: Pearson’s correlation
R^2^: R-squared
SCHFI-V6: The self-Care of Heart Failure Index Version 6.2
SD: Standard deviation
SE: Standard error
t: T-test
