# Supplementary material for: Self-care behavior: a new insight of the role of impulsivity into decision making process in persons with heart failure
Source: BMC Cardiovasc Disord. 2020 Jul 27;20:349. doi: 10.1186/s12872-020-01617-8 (PMC7385854; doi:10.1186/s12872-020-01617-8)
Supplement: Supplementary file 1 — Additional file 1. Simple demographic questionnaire was developed by the authors of this study to collect basic demographic data of the participants. [file 12872_2020_1617_MOESM1_ESM.docx]

**Demographics Questionnaire**

Age? _______________ years

ID #: _____________

What is your gender? Male

Female

What is the highest level of education you reached?

Did not complete high school

High school diploma

Vocational or some college

College

Are you smoker? No

Yes

If you smoke, how long have you been smoking?

………………….. (years) and ………………. months

If you smoke, what type do you use?

Cigarettes, ………………… /day

Cigar, ……………………… /day

Electronic cigarettes, …………………. hour/day

Hookah/shishah, ……………………… hour/day

Other,………………………………….

Do you have any allergies? No

Yes

What causes your allergies?

Foods, including ………………………………….

Medications, including ……………………………

Drinks, including…………………………………..

Other, ………………………………………………

Do you have any chronic diseases? No

Yes, name the

disease(s)……………………
